# *Gordonia bronchialis*: an emerging opportunistic pathogen—a case report and comprehensive review

**DOI:** 10.1186/s12879-025-11695-8

**Published:** 2025-10-14

**Authors:** Ting Li, Junting Wang, Zhijie Zhang, Yu Cao, Hailin Xu, Yanqiao Hui, Xiaosong Qin

**Affiliations:** 1 Department of Laboratory Medicine, Shengjing Hospital of China Medical University, Shenyang, China; 2 Liaoning Clinical Research Center for Laboratory Medicine, Shenyang, China; 3 School of Pharmacy, Shenyang Pharmaceutical University, Shenyang, China

**Keywords:** Gordonia bronchialis, Bacteremia, Opportunistic pathogen, Acute myeloid leukemia

## Abstract

**Background:**

*Gordonia bronchialis* (*G. bronchialis*) is an emerging opportunistic pathogen that primarily affects immunocompromised individuals and is often associated with indwelling catheters.

**Case presentation:**

This paper presents a case of central line-associated bloodstream infection caused by *G. bronchialis* in a 15-year-old male with acute myeloid leukemia (AML), highlighting diagnostic challenges and successful treatment with antimicrobial therapy. The isolate was identified as *G. bronchialis* using matrix-assisted laser desorption/ionization-time of flight mass spectrometry (MALDI-TOF MS). Subsequent antimicrobial susceptibility testing revealed it to be susceptible to amikacin, amoxicillin-clavulanate, ceftriaxone, ciprofloxacin, clarithromycin, imipenem, linezolid, minocycline, moxifloxacin, trimethoprim-sulfamethoxazole, tobramycin, cefepime, cefotaxime and doxycycline. A comprehensive literature review of 41 reported cases underscores the diverse clinical manifestations of *G. bronchialis* infections, including bacteremia, sternal wound infections, and osteomyelitis, with a 95.1% cure rate. Diagnostic limitations and the need for advanced microbiological techniques are discussed, emphasizing the importance of clinical awareness in immunocompromised patients.

**Conclusions:**

This review advocates for standardized treatment guidelines and further research into virulence mechanisms and biofilm-disrupting agents to combat this pathogen effectively.

## Introduction

*Gordonia bronchialis* (*G. bronchialis*) is an aerobic, beaded, gram-positive bacillus that exhibits weak acid-fastness and belongs to the diverse Actinomycetota phylum and the family Gordoniaceae [1, 2]. Initially classified as an aerobic actinomycete, this bacterium was characterized by its weakly acid-fast properties and rare branching morphology. While primarily it is isolated from soil and the sputum of patients with pulmonary disease, these bacteria are environmental saprophytes commonly found in soil, water, and dust [3, 4]. Notably, *G. bronchialis* has also been isolated from the saliva of domestic animals, particularly dogs, suggesting a potential zoonotic reservoir [3].

Historically considered a rare contaminant with low pathogenic potential, Gordonia species have increasingly been recongnized as emerging opportunistic pathogens in humans over the past few decades [2, 5]. Infections primarily occur in immunocompromised hosts, especially individuals with hematological malignancies, solid organ transplants, indwelling medical devices, or those undergoing chronic dialysis [5, 6]. The pathogen exhibits a strong tropism for prosthetic material, most commonly presenting as catheter-related bloodstream infections, sternal wound infections following cardiothoracic surgery, and peritonitis in patients on peritoneal dialysis [5–8].

The accurate identification of *G. bronchialis* poses a significant diagnostic challenge for clinical microbiology laboratories. Its slow growth, beaded gram-stain morphology, and weak acid-fastness could lead to its misidentification as more common pathogens like Nocardia species, rapidly growing mycobacteria, or Corynebacteria [9–11]. Conventional biochemical tests often lack the resolution for definitive speciation. Consequently, reliance on advanced microbiological techniques, such as matrix-assisted laser desorption/ionization-time of flight mass spectrometry (MALDI-TOF MS) and 16 S ribosomal RNA (rRNA) gene sequencing, is crucial for correct identification [5, 9, 10]. This diagnostic delay could complicate clinical management and appropriate antimicrobial therapy.

Treatment strategies for *G. bronchialis* infections are not standardized, largely due to the rarity of reported cases. While the organism often demonstrates in vitro susceptibility to a wide range of antibiotics, including beta-lactams, aminoglycosides, fluoroquinolones, and sulfonamides, evidence-based guidelines for optimal antimicrobial choice and treatment duration are lacking [6, 7, 12]. A critical hurdle is the absence of Clinical and Laboratory Standards Institute (CLSI) breakpoints for many antibiotics against Gordonia species, making interpretation of susceptibility results complex [6].

Herein, we report a case of central line-associated bloodstream infection caused by *G. bronchialis* in a young, immunocompromised patient with acute myeloid leukemia (AML), highlighting the diagnostic journey and successful therapeutic outcome. Furthermore, we present a comprehensive review of previously reported cases of *G. bronchialis* infections to summarize the epidemiological characteristics, clinical spectrum, diagnostic methods, antimicrobial susceptibility profiles, and treatment outcomes associated with this elusive pathogen (Table 1). The aim of this report is to enhance clinical awareness and provide a consolidated reference to aid in the diagnosis and management of this emerging infection.

**Table 1:** Case reports of human infections caused by *G. bronchialis*

Year	Age/Sex	Infection Type	Site of Isolation	Diagnosis	Underlying Condition	Diagnostic Method	Antimicrobial Treatment	Duration (weeks)	Outcome	Reference
2022	13 F	Abdominal infection	Peritoneal fluid	Peritonitis	Chronic peritoneal dialysis	16 S rRNA sequencing	Vancomycin + ciprofloxacin	5	Cured	[8]
2004	58 F	Bacteremia	Blood	Sequestrated lung	Diabetes mellitus	16 S rRNA sequencing, high-performance liquid chromatography	Vancomycin + ceftriaxone → AMX-CLAV	30	Cured	[13]
2011	52 F	Bacteremia	Blood, catheter tip	Febrile neutropenia	Hodgkin’s lymphoma	16 S rRNA sequencing	TMP-SMX + imipenem-cilastatin	12	Cured	[12]
2013	67 F	Bacteremia	Blood	Herpes simplex virus 1 encephalitis	Diabetes mellitus, autoimmune thyroiditis	16 S rRNA sequencing	Cefepime, vancomycin, piperacillin-tazobactam, cefazolin	2	Cured	[14]
2022	56 F	Bacteremia	Blood, catheter tip	Febrile neutropenia	Burkitt lymphoma	16 S rRNA sequencing, MALDI-TOF MS	TMP-SMX + imipenem	4	Cured	[7]
2023	46 M	Bacteremia	Blood	Bacteremia	AML (relapse)	MALDI-TOF MS	Ceftriaxone	2	Cured	[6]
2025	15 F	Bacteremia	Blood, catheter tip	Febrile neutropenia	AML	MALDI-TOF MS	Cefoperazone-sulbactam → ceftazidime → meropenem + vancomycin	4	Cured	This case
2016	50 F	Cutaneous infection	Abscess aspirate	Abscess	Prior needle injection	16 S rRNA sequencing	AMX-CLAV	2	Cured	[15]
2019	61 F	Cutaneous infection	Lower extremity	Postacupuncture infection	None	16 S rRNA sequencing	Cefpodoxime proxetil	6	Cured	[16]
2022	86 M	Cutaneous infection	Forearm	Posttraumatic infection	Steroid atrophy (psoriasis)	Histopathology	AMX-CLAV→ TMP-SMX→ linezolid	>12	Cured	[17]
2014	92 M	Endocarditis	Pacemaker	Pacemaker-induced endocarditis	Pacemaker implantationm, hypertension, pulmonary embolism	16 S rRNA, rpoB sequencing	Amoxicillin	7	Cured	[10]
2022	88 F	Endocarditis	Blood, pacemaker leads	Vegetations on echocardiogram	Atrial fibrillation	MALDI-TOF MS, 16 S rRNA sequencing	Ceftriaxone + ciprofloxacin	6	Cured	[18]
2019	63 F	Endophthalmitis	Vitreous fluid	Chronic uveitis	Intraocular lens implant, COPD, diabetes	16 S rRNA sequencing, MALDI-TOF MS	Amikacin → ceftazoline + moxifloxacin	3	Partial recovery	[19]
2024	35 M	Endophthalmitis	Vitreous fluid	Chronic endophthalmitis	Intraocular collamer lens implantation	mNGS	Vancomycin, ceftriaxone, levofloxacin, tobramycin-dexamethasone	5	Cured	[20]
2014	70 M	Peritonitis	Peritoneal fluid	PD-associated peritonitis	Continuous ambulatory peritoneal dialysis, diabetes	Not specified (likely 16 S rRNA sequencing)	Vancomycin → imipenem + amikacin	2	Cured	[21]
2015	64 F	Peritonitis	Peritoneal fluid	PD-associated peritonitis	Hypertension	16 S rRNA and secA1 sequencing, MALDI-TOF MS	Cefazolin + gentamicin → meropenem + amikacin→ meropenem→ levofloxacin	7	Cured	[22]
2018	32 M	Peritonitis	Peritoneal fluid	PD-associated peritonitis	Focal segmental glomerulosclerosis	MALDI-TOF MS, 16 S rRNA sequencing	Vancomycin → ciprofloxacin	5	Cured	[23]
2022	68 M	Pneumonia	Bronchial aspirate	Foreign body aspiration	Intraductal pancreatic neoplasm	16 S rRNA sequencing	No antibiotics	N/A	Cured	[24]
2022	70 M	Pneumonia	Sputum	Pneumonia	COPD	MALDI-TOF MS	TMP-SMX	N/A	Cured	[9]
2023	78 M	Pneumonia	Bronchoalveolar lavage fluid	Pneumonia	Combined pulmonary fibrosis and emphysema, small cell lung carcinoma (stage 4)	MALDI-TOF MS, 16 S rRNA sequencing	AMX-CLAV	1	Worsened (palliative care)	[25]
2024	35 F	Postblepharoplasty infection	Eyelid cyst	Postblepharoplasty infection	Postblepharoplasty	Histopathology	AMX-CLAV	8	Cured	[26]
2005	43 F	Recurrent breast abscess	Breast tissue	Spontaneous abscess	None	16 S rRNA sequencing	Penicillin + flucloxacillin → AMX-CLAV + metronidazole → doxycycline + clindamycin → doxycycline	20	Cured	[27]
2013	76 F	Sternal osteomyelitis	Sternal wound	Post-CABG infection	None	16 S rRNA sequencing	Ceftriaxone + ciprofloxacin	5	Cured	[28]
2014	69 F	Sternal osteomyelitis	Sternal bone	Post-CABG infection	Postcardiac surgery, diabetes mellitus	Biochemical, 16 S rRNA sequencing, MALDI-TOF MS	Vancomycin + cefotetan → penicillin G → imipenem	8	Cured	[29]
2017	69 M	Sternal osteomyelitis	Sternal wound	Post-CABG infection	Post-CABG surgery, diabetes mellitus	MALDI-TOF MS, 16 S rRNA sequencing	Vancomycin → Ceftazoline	8	Cured	[30]
2019	74 M	Sternal osteomyelitis	Sternal wound	Post-CABG infection	Post-CABG, hypertension, asthma	MALDI-TOF MS, 16 S rRNA sequencing	Vancomycin + meropenem	N/A	Cured	[3]
2022	81 M	Sternal osteomyelitis	Sternal bone, tissue	Osteomyelitis	CAD, hypertension, diabetes mellitus	16 S rRNA and hsp65 sequencing	Ceftriaxone → ampicillin	12	Cured	[31]
1991	51–68 M	Sternal wound infection (7 cases)	Sternal wound	Post-CABG infection	CAD	Biochemical tests, mycolic acid analysis	Ciprofloxacin, TMP-SMX, ceftriaxone	5–15	Cured	[32]
2012	56–80 M	Sternal wound infection (3 cases)	Sternal wound	Post-CABG infection	Post-CABG	16 S rRNA sequencing	Imipenem, linezolid	6	Cured	[33]
2016	64 F	Sternal wound infection	Sternal wound	Sternal wound infection	Postvalve replacement, rheumatic heart disease	MALDI-TOF MS, 16 S rRNA sequencing	Clindamycin + ceftazoline → imipenem + ciprofloxacin → teicoplanin + rifampin	10	Cured	[5]
2022	26 F	Sternal wound infection	Breast tissue	Postreduction mammoplasty	None	MALDI-TOF MS	AMX-CLAV	6	Cured	[34]
2024	58 M	Sternal wound infection	Sternal wound	Sternal wound infection	Post-CABG, diabetes mellitus	MALDI-TOF MS	Linezolid → cefpodoxime	5	Cured	[35]
2012	22 F	Tibial osteomyelitis	Bone tissue	Postarthroscopy infection	None	16 S rRNA sequencing	TMP-SMX → AMX-CLAV → vancomycin → ciprofloxacin	6	Cured	[36]

*Abbreviations*: *AML* acute myeloid leukemia, *AMX-CLAV* amoxicillin‒clavulanate, *CABG* coronary artery bypass graft, *CAD* coronary artery disease, *COPD* chronic obstructive pulmonary disease, *F* female, *M* male, *PD* peritoneal dialysis, *TMP‒SMX* trimethoprim‒sulfamethoxazole

## Case presentation

**Fig. 1:**
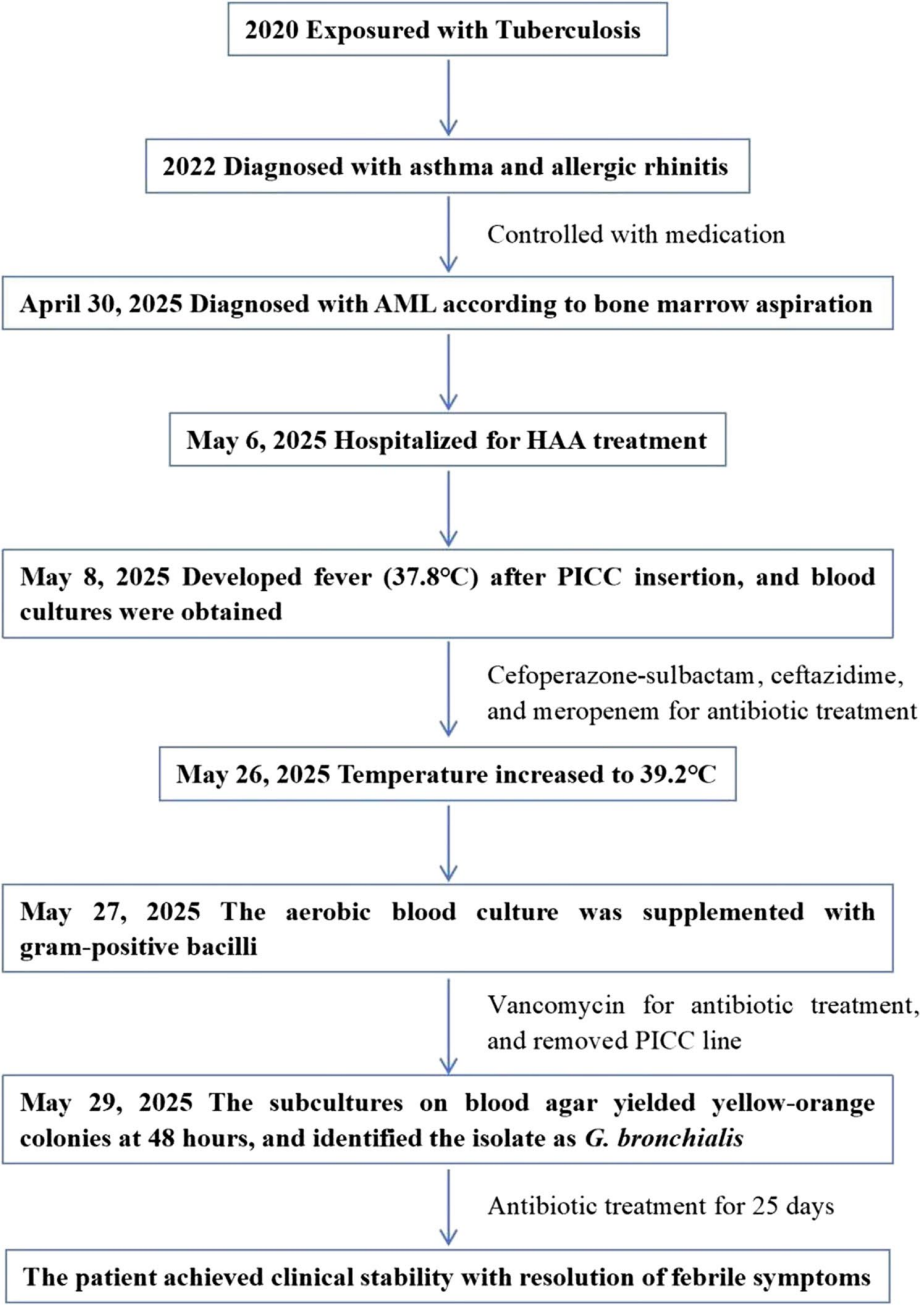
Flow chart of the development process and outcome of the case. Abbreviation: AML, acute myeloid leukemia; HAA, a chemotherapy regimen that consists of drugs Homoharringtonine, cytarabine and aclarubicin; PICC, peripherally inserted central catheter

A 15-year-old male was admitted to our department due to discontinuous fever for one month (Fig. 1). One month prior, the patient had undergone bone marrow aspiration and empirical antimicrobial therapy (imipenem) at an outside hospital, where findings were highly suggestive of AML.

### Medical history

#### Tuberculosis exposure

Close contact with a brother diagnosed with pulmonary tuberculosis in 2020 (since recovery).

#### Respiratory comorbidities

Diagnosed with asthma and allergic rhinitis in 2022, well controlled with fluticasone nasal spray and budesonide/formoterol (Symbicort).

#### Drug allergy

Penicillin hypersensitivity (confirmed by a positive skin test).

### Hospital course

Following peripherally inserted central catheter (PICC) insertion after admission, the patient developed fever (37.8 °C) on the third hospital day, and paired blood fungal and bacterial cultures (catheter and peripheral) were obtained. Rapid plasma reagin, T-SPOT. TB, human immunodeficiency virus, rheumatoid factor, immunoglobulin, and urine tests were normal.

Empirical therapy with IV cefoperazone-sulbactam (3 g q12h) was initiated but failed to resolve the discontinuous fever, changing to IV ceftazidime (2 g q12h) and later IV meropenem (1 g q8h). Following the initial treatment, the patient’s temperature increased to 39.2 °C.

**Fig. 2:**
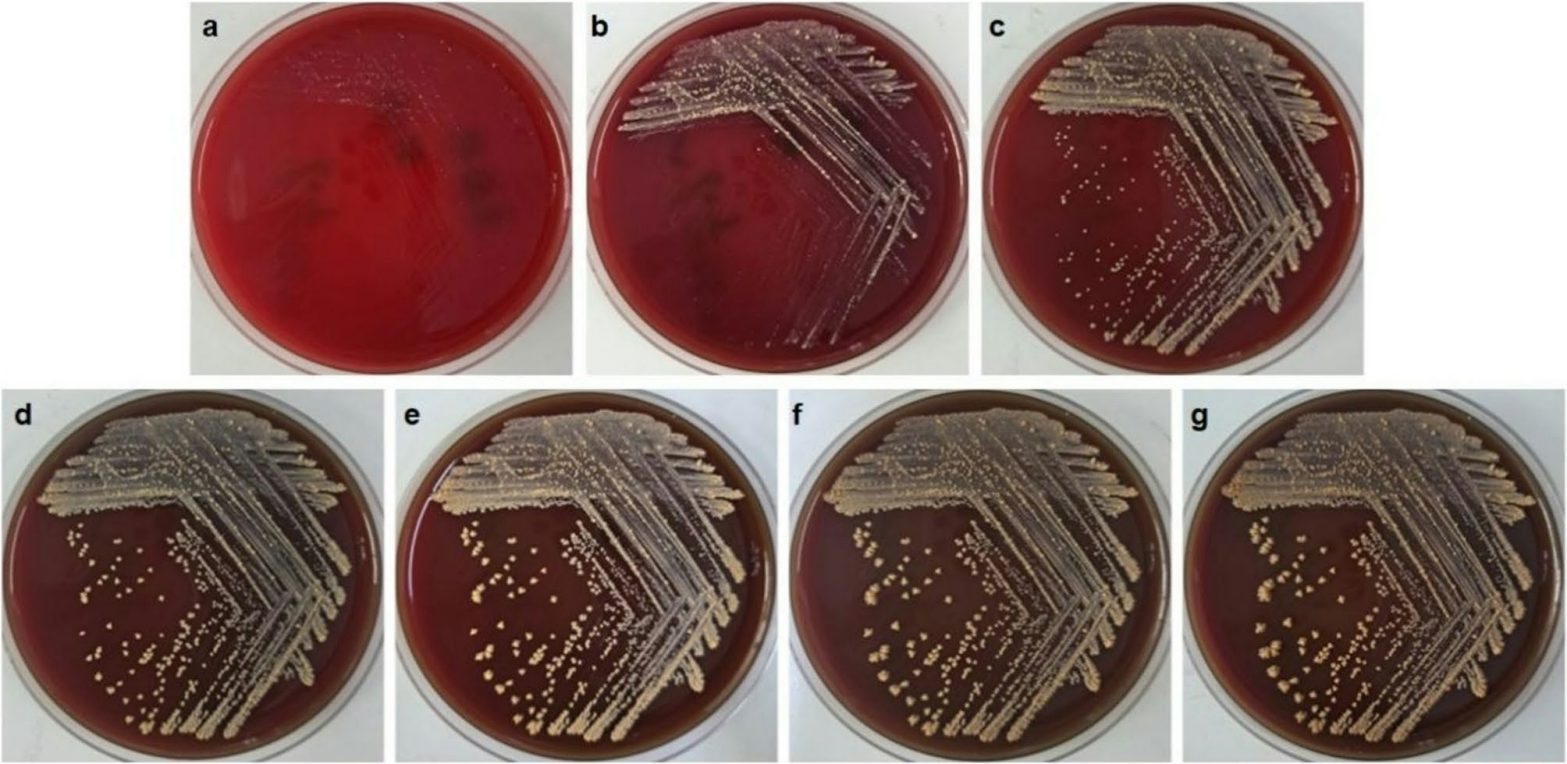
Different incubation times for the colony growth characteristics of *G. bronchialis* subcultured on blood agar: 24 h (**a**), 48 h (**b**), 72 h (**c**), 96 h (**d**), 120 h (**e**), 144 h (**f**), and 168 h (**g**)

**Fig. 3:**
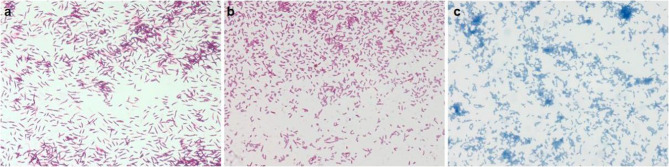
Microscopic morphological characteristics of smears (magnification: 1000×): Gram staining (**a**), modified Ziehl–Neelsen staining (**b**), and Ziehl–Neelsen staining (**c**)

Simultaneously, the aerobic blood fungal culture bottle drawn (catheter line) was supplemented with gram-positive bacilli (after 18 days of incubation), and IV vancomycin (1 g every 12 h) was added empirically. With this positive culture, it was decided to remove his PICC line. The subcultures on blood agar yielded yellow‒orange colonies at 48 h (Fig. 2). Gram staining revealed beaded gram-positive bacilli, while modified Ziehl–Neelsen staining demonstrated slight acid fastness (Fig. 3). Conventional Ziehl–Neelsen staining was negative. We identified the isolate as *G. bronchialis* (99.9% confidence) via MALDI-TOF MS (BioMérieux). However, other blood cultures did not reveal any organisms.

Antimicrobial susceptibility testing was performed via the minimum inhibitory concentration (MIC) test strip method (Autobio; Bio-Kont). The antimicrobial agents tested included amikacin, amoxicillin-clavulanate, ceftriaxone, ciprofloxacin, clarithromycin, imipenem, linezolid, minocycline, moxifloxacin, trimethoprim-sulfamethoxazole, tobramycin, cefepime, cefotaxime, doxycycline, meropenem, penicillin and vancomycin. The CLSI (M45-A3) breakpoints for actinomycetes were used. The MIC values were as follows (Table 2): 0.125 mg/L for amikacin, 0.016 mg/L for amoxicillin-clavulanate, 0.125 mg/L for ceftriaxone, 0.032 mg/L for ciprofloxacin, 0.5 mg/L for clarithromycin, 0.006 mg/L for imipenem, 0.75 mg/L for linezolid, 0.5 mg/L for minocycline, 0.008 mg/L for moxifloxacin, 0.016 mg/L for trimethoprim-sulfamethoxazole, 0.125 mg/L for tobramycin, 0.25 mg/L for cefepime, 0.125 mg/L for cefotaxime, 0.25 mg/L for doxycycline, 0.19 mg/L for meropenem, 0.125 mg/L for penicillin and 0.38 mg/L for vancomycin. *G. bronchialis* is susceptible to these antimicrobial agents, while CLSI guidelines lack MIC breakpoints for meropenem, penicillin and vancomycin (likely due to insufficient clinical data, the absence of standardized testing methods, and/or limited evidence of in vivo activity).

**Table 2:** Susceptibility of *G. bronchialis* isolates to antimicrobial agents

Antibiotic	MIC (mg/L)	Interpretation
Amikacin	0.125	S
Amoxicillin-clavulanate	0.016	S
Ceftriaxone	0.125	S
Ciprofloxacin	0.032	S
Clarithromycin	0.5	S
Imipenem	0.006	S
Linezolid	0.75	S
Minocycline	0.5	S
Moxifloxacin	0.008	S
Trimethoprim-sulfamethoxazole	0.016	S
Tobramycin	0.125	S
Cefepime	0.25	S
Cefotaxime	0.125	S
Doxycycline	0.25	S
Meropenem	0.19	N/A
Penicillin	0.125	N/A
Vancomycin	0.38	N/A

(*R* Resistant, *S* Susceptible, *I* Intermediate)

### Outcome

Following a 25-day course of concurrent antimicrobial therapy and chemotherapy, the patient achieved clinical stability with resolution of febrile symptoms. Markers of systemic inflammation significantly improved, with rapid C-reactive protein levels declining from a peak of 106 mg/L (indicating severe systemic inflammation) to 22 mg/L (within the near-normal range). The patient was subsequently discharged in stable condition.

## Discussion

*Gordonia* spp. are weakly acid-fast, rod-shaped bacteria that form cord-like colonies; these bacteria were discovered from soil in 1971 and were subsequently isolated from the sputum of tuberculosis patients [4]. The *G. bronchialis* strain isolated from our patient represents the first instance of *Gordonia spp.* recovered in our laboratory. The patient was a 15-year-old male with AML reported with a central line-associated *G. bronchialis* bacteremia. His history of close contact with a tuberculosis patient, which not definitively proven as the source, presents a plausible and previously suggested zoonotic or environmental transmission route that is often difficult to establish but is crucial for understanding epidemiology

A critical novel aspect of our report is the detailed documentation of isolate’s extensive antimicrobial susceptibility profile using the MIC test strip method. We have provided specific MIC values for 17 antimicrobial agents, revealing remarkably low MICs for a wide range of antibiotics, including amikacin (0.125 mg/L), imipenem (0.006 mg/L), and moxifloxacin (0.008 mg/L). This comprehensive susceptibility data is a valuable addition to the literature, as breakpoints for many of these drugs against *Gordonia spp.* are not defined by CLSI due to a lack of evidence. Our findings have robustly demonstrated in vitro susceptibility to multiple drug classes, which expanded the potential therapeutic options beyond the empirically used vancomycin. It is particularly relevant given the patient’s penicillin allergy, which often limits treatment choices. Furthermore, the successful resolution of infection with a combination of meropenem and vancomycin, followed by the patient’s clinical stabilization concurrent with chemotherapy, has provided a real-world example of effective management in a complex clinical scenario.

Our comprehensive literature review, synthesizing 41 *G. bronchialis* infection cases from 1991 to 2025 (based on PubMed case reports), allows us to contextualize these findings on a larger scale. Among 6 cases of bacteremia, 4 cases (66.7%, Table 1) were catheter-related infections, all of which occurred in patients with hematologic malignancies (2 cases of AML and 2 cases of lymphoma) [6, 8, 11]. In addition to bacteremia, *G. bronchialis* can cause various clinical infection types, including sternal wound infection (31.7%), sternal osteomyelitis (12.2%), cutaneous infection (7.3%), peritonitis (7.3%), pneumonia (7.3%), endocarditis (4.9%), endophthalmitis (4.9%), postblepharoplasty infection (2.4%), recurrent breast abscess (2.4%), abdominal infection (2.4%) and tibial osteomyelitis (2.4%) (Table 1). Although the global increase in the number of *G. bronchialis* infection cases raises vigilance for this emerging opportunistic threat, our calculated high cure rate of 95.1% (39/41) reinforces that, despite diagnostic challenges, appropriate interventions often result in good outcomes. However, our analysis also highlighted a significant knowledge gap: the absence of standardized treatment guidelines. The current practice heavily relied on vancomycin (22.0% of cases), yet our susceptibility data and other cases have shown high efficacy for other agents like carbapenems, amoxicillin-clavulanate, and fluoroquinolones. This dissonance between practice and evidence underscored our study’s called for the development of evidence-based guidelines to streamline therapy and potentially avoid the overuse of glycopeptides.

Notably, *G. bronchialis* has mixed characteristics of both Mycobacterium and Nocardia species [4, 37]. Conventional biochemical tests frequently misclassify *G. bronchialis* as Nocardia or nontuberculous mycobacteria. This case exemplified the paramount importance of advanced diagnostic techniques like MALDI-TOF MS in correctly identifying this pathogen. The 18-day incubation period required for the blood culture to signal positive was a stark reminder of the patience and suspicion required from clinical microbiologists. In addition, this organism could be identified via other advanced nonphenotypic methods, such as 16 S rRNA sequencing (61.0% of cases) or metagenomic next-generation sequencing (mNGS) (2.4% of cases). Therefore, the novel contribution of this study is multifaceted: it expands the understanding epidemiology of *G. bronchialis* infections, provides crucial in vitro susceptibility data to guide therapy, leverages a large-scale review to confirm excellent prognosis with treatment, and powerfully advocates for heightened clinical awareness and the urgent development of standardized diagnostic and therapeutic protocols to manage this emerging pathogen effectively.

## Conclusions

In conclusion, we report a novel case of *G. bronchialis* bacteremia in a pediatric AML patient, highlighting the critical diagnostic clue of an unusually prolonged 18-day blood culture incubation period. Coupled with the most extensive literature review to date, our findings provide a detailed antimicrobial susceptibility benchmark and establish a high overall cure rate of 95.1% for these infections. *G. bronchialis* is an emerging opportunistic pathogen that demands heightened clinical suspicion in immunocompromised patients with medical devices. Overcoming diagnostic limitations through advanced techniques and moving towards standardized treatment guidelines are essential steps to improving outcomes against this elusive but treatable pathogen.

## Data Availability

The datasets used and/or analyzed during the current study are available from the corresponding author upon reasonable request.

## Abbreviations

AML: Acute myeloid leukemia
CLSI: Clinical and Laboratory Standards Institute
*G.**bronchialis*: *Gordonia bronchialis*
MALDI-TOF MS: Matrix-assisted laser desorption/ionization-time of flight mass spectrometry
MIC: Minimum inhibitory concentration
PICC: Peripherally inserted central catheter
